# Recommendation and intake of dietary supplements periconceptional and during pregnancy: results of a nationwide survey of gynaecologists

**DOI:** 10.1007/s00404-023-07167-6

**Published:** 2023-09-16

**Authors:** Kai J. Buhling, Marie Scheuer, Elena Laakmann

**Affiliations:** 1 Department of Gynecological Endocrinology, University Medical Center Hamburg-Eppendorf, Martinistrasse 52, 20246 Hamburg, Germany; 2 Department of Gynecology, University Medical Center Hamburg-Eppendorf, Martinistrasse 52, 20246 Hamburg, Germany

**Keywords:** Micronutrients, DHA, Fertility, Folic acid, Iodine, Fertility treatment, IVF

## Abstract

**Background:**

Micronutrient supplementation during pregnancy is a controversial issue. For some micronutrients, for example folic acid or iodine, the evidence regarding supplementation is clear, whereas for others, such as zinc or vitamin E, it is not. Studies show that a large number of pregnant women have deficient levels of folic acid and iodine. However, especially with folic acid, starting supplementation during the preconception period is crucial. It is, therefore, important that gynaecologists explain this to their pregnant or preconceptional patients. Our goal was to find out how gynaecologists make their recommendations on this topic, how they assess the compliance of their patients and which micronutrients they consider to be important before/during pregnancy and during breastfeeding.

**Method and Results:**

We sent about 12,000 questionnaires to all registered resident gynaecologists in Germany, with a response rate of 12.2%. Regarding which micronutrients gynaecologists  consider to be particularly important during pregnancy, there was a broad agreement for both folic acid and iodine (> 88% answered yes). According to the questionnaire, doctors rate other micronutrients, such as vitamin D and omega-3 fatty acids, as less essential. The controversial evidence level for many micronutrients certainly plays a role here. Overall, the intake rate, especially for preconceptional women, is classified as rather low (< 60%). The most widely valued reason is the high price of dietary supplements. It was also noticeable that doctors consider certain micronutrients to be particularly important but then do not include them in the products they recommend.

**Conclusion:**

Overall, there seems to be uncertainty about micronutrients in pregnancy and their supplementation. The study situation is often ambiguous and there are no official guidelines, leading to ambiguous recommendations from doctors and therefore low intake rates for pregnant or preconceptional women.

**Supplementary Information:**

The online version contains supplementary material available at 10.1007/s00404-023-07167-6.

## What does this study add to the clinical work

**Table:** 

Preconceptional use of micronutrients is overestimated. Knowledge about the usefulness of micronutrients is heterogeneous. Necessity for guidelines of the societies and more material for HCP and patients.	

## Introduction

Lack of micronutrients before and during pregnancy can lead to congenital malformations such as neural tube defects, cleft lip and heart malformations [1], but the risk can be lowered by supplementation with folic acid [2] Additionally, the pregnancy rate can be elevated by supplementation with iodine, folic acid and omega-3 fatty acids [3].

The current study situation of which micronutrients to take before, during and after pregnancy is sometimes not clear. Furthermore, supplements often differ in their composition; therefore, it is necessary to establish which micronutrients the doctors pay particular attention to during/before pregnancy and during breastfeeding (Table 1)

**Table 1:** Proportion of gynaecologists who consider specific micronutrients to be particularly important in certain stages of pregnancy

	Preconception (%)	During pregnancy (%)	Lactation period (%)
Folic acid	95.3	92.8	61.9
Iodine	75.5	88.4	80.7
Vitamin D	56.7	66.5	56.9
Vitamin E	15.1	19.4	17
Zinc	20.1	24.3	20.3
Omega-3 fatty acids	26.2	70.9	41

This study used a nationwide survey to assess the perspective of gynaecologists in private practise, who have the main responsibility for the prenatal care of pregnant women in Germany.

## Materials and methods

### Population and sample

In June 2019, we sent a self-administered, anonymous questionnaire to all 11,902 registered gynaecologists working in private practise in Germany. The questionnaires were delivered by post and were to be returned within 2 weeks by fax or e-mail.

In addition to some personal data (age, gender, duration of work as a gynaecologist), the questionnaire included nine multiple-choice questions and two tables (see appendix). In one table, the gynaecologists were asked which micronutrients they considered to be important during pregnancy, lactation and preconception. In the other table, the gynaecologists were asked which products they recommended and which products they would choose for themselves.

### Data analyses

Data were entered and analysed using SPSS Statistics version 26.0. For categorical information (e.g. gender, age), we resorted to the Chi-squared test to evaluate associations between variables. We used primary descriptive statistics [4].

## Results

By the end of June 2019, we had received 1454 of the 11,898 questionnaires via fax or e-mail, giving an overall response rate of 12.2%. Of the responding gynaecologists, 79.8% were female and 20.2% were male. The female gynaecologists answered in a higher proportion than the male gynaecologists: 1145/8112 (14%) versus 289/3,794 (7.6%).

The mean age of the respondents was 53.4 ± 8.2 years. On average, the men’s age was 58.6 ± 9.1 years and that of the women was 52.0 ± 7.37 years.

The average work experience as a doctor in private practise was 15.6 ± 9.24 years (males: 20.3 ± 9.55; females: 14.4 ± 8.73).

### Recommendation of periconceptional use of micronutrients

The recommendation of different micronutrients in specific stages of pregnancy (e.g. preconception, pregnancy and lactation) was evaluated.

Nearly all gynaecologists considered folic acid to be important before and during pregnancy, whereas only two-thirds recommended folic acid to be taken during lactation. Iodine was recommended by more than three-quarters of gynaecologists for all three specified stages. On the other hand, only 56–67% of gynaecologists recommended vitamin D supplementation.

An absolute minority (under a fifth) of gynaecologists considered vitamin E to be important before, during and after pregnancy. With regard to zinc, only a quarter of gynaecologists considered any benefit. The considered importance of omega-3 fatty acid supplementation was widely variable: very low in fertility treatment (26.2 %), higher during pregnancy (70.9%) and lower during breastfeeding (41%).

### Intake of dietary supplements before and during pregnancy

To achieve the optimal effect of a sufficient folic acid level in the blood, the intake must be started during preconception [1]. Therefore, gynaecologists need to estimate the percentage of pregnant women taking folic acid or dietary supplements and how many of them started before the pregnancy.

Approximately 60% of gynaecologists think that almost all (80–100%) of their pregnant patients take supplements, whereas less than 15% think that only half of their patients take supplements during pregnancy.

Only 30% of gynaecologists believe that the intake rate of dietary supplements before pregnancy is more than 60%, whereas less than 10% believe that dietary supplements are taken by the majority of patients (80–100%) before pregnancy.

Only 3% of gynaecologists believe that side effects could cause women not to take supplements. However, three-quarters think that the high cost is responsible for any indecision.

Half of gynaecologists believe that their colleagues do not enlighten their patients enough and that the pregnant women therefore do not take supplements.

### Uncertainty about the micronutrients contained in dietary products

There are many products for dietary supplements in pregnancy on the market. These products contain different micronutrients in different dosages. Thus, to perform an advisory function, one has to know the composition of these products.

In a second table, gynaecologists were asked to indicate which product they would recommend or take themselves.

It has already been evaluated whether the micronutrients identified as most important in the first table (Table 1) are actually included in the vitamin/micronutrients products recommended by the gynaecologists. For example, over 50% of gynaecologists who consider omega-3 fatty acids to be particularly important during pregnancy recommended products that do not contain them in the first stage (e.g. Elevit^®^, Femibion^®^). Furthermore, almost half of those who consider vitamin D in pregnancy to be important recommended a product containing only 5 µg of vitamin D; however, with insufficient endogenous synthesis, 20 µg/day are recommended during pregnancy and lactation (German Nutrition Society, 2012). The same applies to vitamin E: 50% of those who consider vitamin E to be important during pregnancy recommended a product that does not contain vitamin E at all.

### The patients’ role in the decision-making process

Finally, gynaecologists have an advisory role, giving recommendations but not prescribing products. Therefore, it is up to the patient to decide whether, from when and for how long they want to take supplements.

The evaluation of the questionnaire revealed that half of the gynaecologists were asked by the patients themselves concerning the nutritional supplements. Also, 75% of gynaecologists indicated that dietary supplements have become more relevant in recent years.

It can, therefore, be assumed that women seek advice from friends or are influenced by advertising or by social media rather than the gynaecologist’s recommendation.

## Discussion

A similar study was conducted in 2000 by Power et al., but with a focus on folic acid substitution, and 488 questionnaires were answered [3].

This study, with over 1400 questionnaires answered, appears to be the largest study to survey gynaecologists on the topic of dietary supplements in pregnancy. This study shows the lack of guidelines about preconceptional supplementation for gynaecologists.

Our survey detected that there is no agreement regarding dietary supplements in/before pregnancy or during breastfeeding amongst gynaecologists. There are many supplement products with different compositions, the evidence for the supplementation of some micronutrients is not clear, and, according to observation of the gynaecologists, not all pregnant women take dietary supplements.

### Necessity for taking micronutrients during pregnancy

#### Folic acid

The evidence for the intake for folic acid before and during the pregnancy is clear. Adequate intake of folic acid leads to a significantly reduced occurrence of spina bifida and congenital heart defects [4]. However, the neural tube closes at the beginning of pregnancy (4th week of pregnancy), so the intake of folic acid must be started during preconception [5]. This is a safer way to achieve adequate folic acid levels and preventive benefits at the beginning of pregnancy.

Therefore, gynaecologists should regularly ask their patients about the desire to have children and, if necessary, inform them about the need to take folic acid.

A preconceptional intake of folic acid (400 µg), which should be continued especially at the beginning of pregnancy, is therefore generally recommended [6]. The gynaecologists in our survey also agreed (preconception: 95.3%; during pregnancy: 92.8%).

#### Iodine

Three-quarters of gynaecologists agreed on the need for a sufficient level of iodine in the blood. The evidence concerning the supplementation of iodine is also clear. Iodine deficiency is one of the most common reasons for manifest or latent hypothyroidism [7]. During pregnancy, the iodine requirement for adequate thyroid function also increases [8]. Studies show that hypothyroidism in pregnant women is associated with subtle delays in newborn development during follow-up examinations [9]. In addition, the foetus itself produces its thyroid hormones from the 20th week of pregnancy and requires a sufficient amount of iodine for this.

It is therefore recommended that, in addition to a balanced diet, an iodine preparation (100–150 µg) should be taken, at a dose corresponding to the low to middle of the range (100–200 μg/d) specified in the maternity guidelines and regarded as safe for iodine supplementation during pregnancy.

#### Vitamin D

A sufficient maternal serum level of vitamin D is essential for the child's bone mineralization [10]. Several studies showed the association of low maternal vitamin D status with pregnancy complications such as preeclampsia, preterm birth or low birthweight [11]. However, studies have also shown that 18–80% of pregnant women have deficient vitamin D levels [12]. In this case, a daily vitamin D intake of 20 µg is recommended [2]. In addition, further studies showed that women who are undergoing in vitro fertilisation should take vitamin D due to its positive effect on the endometrium [13]. Another study showed that women with higher vitamin D levels during IVF had a higher pregnancy rate [14]. This is also mentioned in the guidelines of the German Society for Gynaecology and Obstetrics for diagnostics and therapy prior to assisted reproductive medicine treatment [15]. In addition, studies suggest that there is an association between prenatal vitamin D deficiency and autism spectrum disorder [16]. When vitamin D synthesis is reduced (particularly in the winter months), an adequate vitamin D level can only be provided by diet intake. Pregnant women with little sun exposure or with dark skin are recommended to take a vitamin D supplement. According to the survey, 58–67% of gynaecologists seem to be aware of this. Of course, vitamin D supplementation is not necessary for every woman, but it should be clarified, especially in the winter months, about the widespread vitamin D deficiency and its possible consequences, with recommendations given accordingly.

#### Vitamin E

There is an evidence of a possibility to cover the daily vitamin E intake through a diet. Vitamin E deficiency is rare in healthy adults. However, an insufficient intake of vitamin E during pregnancy can lead to complications, such as premature placental abruption or preeclampsia [17]. Devereux et al. showed that lower maternal vitamin D and E intake during pregnancy is associated with increased risk of the child wheezing and being diagnosed with asthma in the first 10 years [18]. Thus, the Vitamin E supplementation can be beneficial during pregnancy. However, there no general recommendation can be done. This aspect corresponds to the results of our survey: only one-fifth of gynaecologists think that vitamin E is particularly important before/during/after pregnancy. In the future, further studies should clarify the role of vitamin E supplementation during pregnancy.

#### Zinc

Similar to vitamin E, there are no clinical studies showing the benefits of zinc supplementation in pregnancy, but approximately 18% of pregnant women are zinc deficient and a slight additional need is assumed during pregnancy; therefore, zinc supplementation could be beneficial. 11–13 mg/d is the recommended dose of zinc for pregnant women [19].

According to the questionnaire, only 25% of gynaecologists represent the opinion that zinc supplementation is necessary. Therefore, further studies are needed to clarify whether zinc deficiency has an impact on pregnancy.

#### Omega-3 fatty acid

DHA is an essential omega-3 fatty acid for building up cell membranes, mainly in the brain and eyes [20]. A sufficient supply can be obtained by a regular consumption of fatty sea fish [21]. For example, vegetarians have a lower DHA intake than non-vegetarians [22]. In randomised-controlled trials, the supplementation of fish oil or long-chain omega-3 fatty acids led to a significant reduction in the risk of premature birth up to 34 weeks of gestation [23]. Furthermore there is an evidence of a reduced risk of perinatal depression under an adequate omega-3 fatty acid supplementation [24]. Chiu et al. examined the relationship between omega-3 fatty acids and the onset of pregnancy. For every 1% increase of DHA in serum, the clinical pregnancy rate increased by 8% [15, 25]. There is also evidence that preconceptional supplementation improves embryonal development [26]. However, the findings on the use of DHA supplements in pregnancy for the cognitive development of the child are inconsistent. Nonetheless, women who do not eat fatty sea fish regularly are advised to take a DHA supplement [27]. 30% of gynaecologists do not seem to be aware of this. Ultimately, there are also several dietary supplement products that do not contain omega-3 fatty acids.

In summary, gynaecologists seem to agree that folic acid and iodine must be given special attention during pregnancy. In contrast, despite clear recommendations, the gynaecologists seem not to be aware of the importance of the supplementation of vitamin D and omega-3 fatty acids. For vitamin E and zinc, future studies should clarify their role during pregnancy.

### Uncertainty about the composition of the different products

There are many different nutritional supplements for pregnant women on the market that have different compositions. There is also the option of adding the micronutrients individually, although it is sometimes not clear whether the products from the chemist contain the necessary dose.

As part of our survey, gynaecologists should indicate which product they recommend most often or even take themselves. We detected that many gynaecologists who consider omega-3 fatty acids to be particularly important end up recommending products that do not contain any. This is also the case for vitamin E.

A possible explanation for this aspect is a big amount of products on the market. Possibly, because of lack of time, the doctors are not always able to deal with the compositions of individual products. At the same time, it is not clear if the doctors are properly informed about the current recommendations on this topic. Although there are no guidelines available, the recommendations concerning the substitution of vitamins and minerals during the pregnancy are existing [27]. In our questionnaire, only half of the doctors stated that they are aware of the current recommendations of the ‘Federal Center for Nutrition’, in which recommendations for food supplements during pregnancy are made.

Some physicians believe that a balanced diet adequately covers the micronutrient requirements of a pregnant woman. However, this is not the case for folic acid, iodine, and Omega-3 fatty acids. Vitamin D is also insufficiently absorbed through a dietary intake and there is a risk of deficiency in case of a low sun exposure. Therefore, dietary intake covers the need only for a part of the micronutrients [26].

Thus, only products that adhere to the relevant recommendations should be offered.

### Low estimated intake of dietary supplements, especially during preconception

**Fig. 1:**
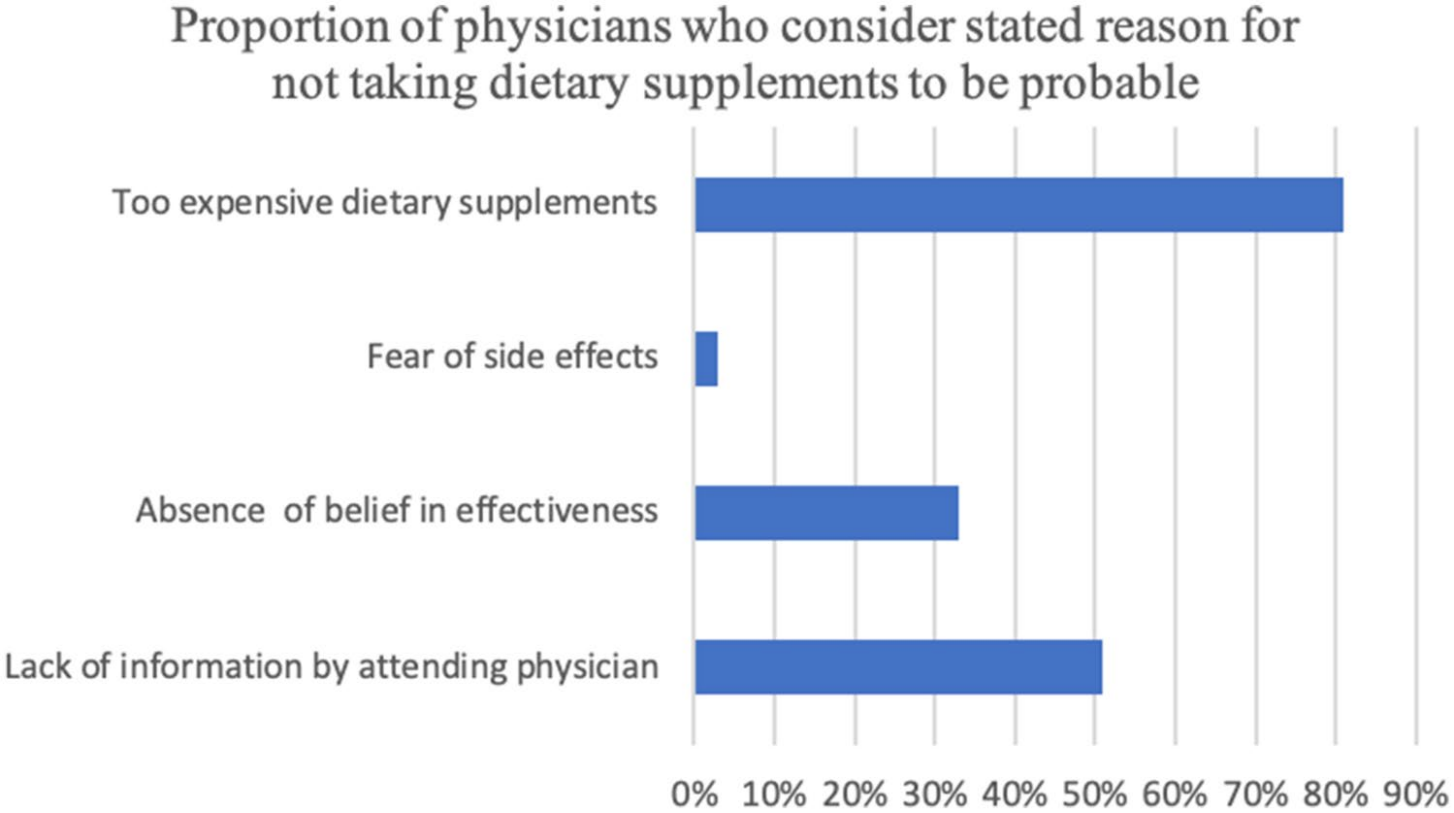
Possible reasons for not taking supplements

The reasons why pregnant women generally do not take nutritional supplements seem to be clear: 75% of doctors say that the reason is a high cost and half of the doctors even say that a lack of information from their colleagues is the reason (Fig. 1).

An important reason for a low preconceptional intake of folic acid might be that pregnancies can occur unplanned. Therefore, it is important to regularly ask women if they want to have children. Our survey showed that 88.5% of doctors talk about this aspect with their patients. The necessity of nutritional supplements should always be explained and recommendations should be made if necessary.

## Conclusions

Ultimately, there is no consensus on all available nutritional supplements during pregnancy. In the case of individual micronutrients, this is due to an inconclusive study situation and also a lack of guidelines and insufficiently known recommendations.

Gynaecologists are mostly aware of the importance of certain micronutrients such as folic acid or iodine, but there are often uncertain regarding omega-3-fatty acids and some micronutrients such as vitamin D and vitamin E. In addition, sometimes products are recommended that do not contain the micronutrients that are actually considered to be important. In the future, guidelines should be formulated to which the product manufacturers must adhere. Until then, it is important as a gynaecologist to examine the composition of the products, so that products are recommended that contain all the relevant micronutrients.

Although a preconceptional intake of certain micronutrients such as folic acid is essential, doctors estimate the intake rate before pregnancy to be low. Nevertheless, this rate could be improved in the future by providing information during the doctor's consultation.

### Supplementary Information

Below is the link to the electronic supplementary material.

Supplementary file1 (PDF 85 kb)

## Data Availability

Not available.
