# Supplementary material for: Recommendation and intake of dietary supplements periconceptional and during pregnancy: results of a nationwide survey of gynaecologists
Source: Arch Gynecol Obstet. 2023 Sep 16;308(6):1863–9. doi: 10.1007/s00404-023-07167-6 (PMC10579106; doi:10.1007/s00404-023-07167-6)
Supplement: Supplementary file 1 — Supplementary file1 (PDF 85 kb) [file 404_2023_7167_MOESM1_ESM.pdf]

Universitätsklinikum Hamburg-Eppendorf

Survey: Dietary supplements during pregnancy

1. How old are you? ………….. years
2. How many years have you been in private practice as a gynecologist?
   …….. years
3. Are you **O** Male or **O** Female?
4. Approximately how many **pregnant** patients in your practice are taking the dietary supplements recommended by you?
   O 0-20% **O** 20-40% **O** 40-60% **O** 60-80% **O** 80-100%
5. Approximately how many of your patients in your practice are taking the dietary supplements recommended by you **before pregnancy**?
   O 0-20% **O** 20-40% **O** 40-60% **O** 60-80% **O** 80-100%
6. What vitamins and micronutrients do you consider for women who wish to have children, during pregnancy or during breastfeeding for especially important?

**vitamins/micronutrients**	Desire to have children	During pregnancy	While breastfeeding
Vit. A			
Vit. B1 (Thiamin)			
Vit. B2 (Riboflavin)			
Pantothenat (Vit. B5)			
Folate (Vit. B9)			
Vitamin B12			
Vitamin C			
Vitamin D			
Vitamin E			
Biotin			
Vitamin K			
Magnesium			
Iron			
Iodine			
Zinc			
Calcium			

Selenium			
Copper			
Manganese			
Omega 3 fatty acids			
Probiotics			
Other:................................			

7. Approximately how many of your pregnant patients proactively approach you inquiring about meaningful dietary supplements in the context of fertility and pregnancy?

   O 0-20% **O** 20-40% **O** 40-60% **O** 60-80% **O** 80-100%

8. Do you perceive that the subject of dietary supplements during pregnancy has gained increased relevance in recent years?

   O Yes. **O** No. **O** I don't know.

9. Do you actively inquire with patients of reproductive age (20-40 years) about their desire to conceive?

   O Yes. **O** No.

10. Do you consider the multi-stage concepts developed by manufacturers (e.g. Femibion®, Elevit®), involving varying compositions of micronutrients in dietary supplements based on the progression of pregnancy, to be meaningful?

    O Yes. **O** No. **O** I don't know.

11. Which dietary supplement products do you know, routinely recommend, or would you take yourself?

	**I am aware.**	**I recommend regularly (>90% of pregnant patients).**	**I would take myself.**
**adfetal®**			
**Elevit®**			
**Femibion®**			
**Folio®**			
**Nestle materna DHA®**			
**Orthomol natal®**			
**Velnatal plus®**			
**...........................**			

12. In your opinion, why do pregnant women or women who wish to have children refrain from taking nutritional supplements?

O lack of information from the doctor

O lack of belief in the effectiveness

O side effects

O too high cost of the dietary supplements

O other: ……………..

13. Could you imagine pharmacies changing the recommendation, resulting in a different supplement being taken than recommended (e.g., due to better availability or higher profit margin)?

O Yes. **O** No.

14. Are you familiar with the dietary guidelines issued by the "Federal Center for Nutrition" in 2018?

O Yes. **O** No.

15. Do you have any other comments about this survey?

…………………………………………………………………………………………………

…………………………………………………………………………………………………

…………………………………………………………………………………………………
